# Characterization of the novel indolylmaleimides' PDA-66 and PDA-377 effect on canine lymphoma cells

**DOI:** 10.18632/oncotarget.9297

**Published:** 2016-05-12

**Authors:** Wen Liu, Julia Beck, Laura C. Schmidt, Catrin Roolf, Anahit Pews-Davtyan, Barbara C. Rütgen, Sabine Hammer, Saskia Willenbrock, Anett Sekora, Arndt Rolfs, Matthias Beller, Bertram Brenig, Ingo Nolte, Christian Junghanss, Ekkehard Schütz, Hugo Murua Escobar

**Affiliations:** ^1^ Department of Medicine, Clinic III - Hematology/Oncology/Palliative Care, Rostock University Medical Center, Rostock, Germany; ^2^ Small Animal Clinic, University of Veterinary Medicine Hannover, Hannover, Germany; ^3^ Chronix Biomedical, Göttingen, Germany; ^4^ Leibniz-Institute for Catalysis at the University of Rostock, Rostock, Germany; ^5^ Clinical Pathology, Department of Pathobiology, University of Veterinary Medicine Vienna, Vienna, Austria; ^6^ Institute of Immunology, Department of Pathobiology, University of Veterinary Medicine Vienna, Vienna, Austria; ^7^ Albrecht-Kossel-Institute for Neuroregeneration (Akos), Center for Mental Health, University of Rostock, Rostock, Germany; ^8^ Centogene AG, Rostock, Germany; ^9^ Institute of Veterinary Medicine, Georg-August-University Göttingen, Göttingen, Germany

**Keywords:** PDA, arylindolylmaleimides, canine lymphoma, transcriptome sequencing

## Abstract

Protein kinase inhibitors are widely used in chemotherapeutic cancer regimens. Maleimide derivatives such as SB-216763 act as GSK-3 inhibitor targeting cell proliferation, cell death and cell cycle progression.

Herein, the two arylindolylmaleimide derivatives PDA-66 and PDA-377 were evaluated as potential chemotherapeutic agents on canine B-cell lymphoma cell lines. Canine lymphoma represents a naturally occurring model closely resembling the human high-grade non-Hodgkin's lymphoma (NHL). PDA-66 showed more pronounced effects on both cell lines. Application of 2.5μM PDA-66 resulted in a significant induction of apoptosis (approx. 11 %), decrease of the metabolic activity (approx. 95 %), anti-proliferative effect (approx. 85 %) and cell death within 48h. Agent induced mode of action was characterized by whole transcriptome sequencing, 12 h and 24 h post-agent exposure. Key PDA-66-modulated pathways identified were cell cycle, DNA replication and p53 signaling. Expression analyses indicated that the drug acting mechanism is mediated through DNA replication and cycle arrest involving the spindle assembly checkpoint.

In conclusion, both PDA derivatives displayed strong anti-proliferation activity in canine B-cell lymphoma cells. The cell and molecular PDA-induced effect characterization and the molecular characterization of the agent acting mechanism provides the basis for further evaluation of a potential drug for canine lymphoma serving as model for human NHL.

## INTRODUCTION

Canine lymphoma represent approx. 24 % of all occurring canine neoplasms and 83 % of all canine hematopoietic neoplasms [1–3]. Compared to human lymphoma, the canine tumor shows a higher incidence with 13-33 cases per year in 100,000 dogs [4]. Concerning tumor development, progression and disease pattern lymphoma in dogs presents highly similar to human high-grade non-Hodgkin's lymphoma (NHL) and thus is considered to serve as a comparative animal model of human NHL [2, 5].

Without treatment, most dogs suffering from malignant lymphoma die within four to six weeks [6]. In dogs, combination chemotherapy protocols are superior in multicentric lymphoma and conventional CHOP-based chemotherapy induces remission in approx. 80 % to 95 % [3]. However, the majority of dogs undergoing chemotherapy suffer disease recurrence within 12 months. Further, the recurrent lymphoma cells are described to be highly resistant to the initial chemotherapeutic protocol [7, 8].

Conventional chemotherapeutic protocols are currently being completed by addition of inhibitory agents targeting specific pathways e.g. GSK-3 or PI3K. These pathways are reported to be altered in different neoplasias such as leukemia [9, 10].

Arylindolylmaleimides are synthetic molecules, consisting of a maleimide group conjugated to a bicyclic indole ring and an additional aromatic or heterocyclic component [11]. PDA-66 and PDA-377 are two arylindolylmaleimides representing analogues of the GSK-3-specific inhibitor SB-216763 characterized by differences in their chemical substitutions [11, 12]. Application of PDA-66 to human acute lymphoblastic leukemia (ALL) cell lines induced significant anti-proliferative effects leading to apoptosis but did not inhibit GSK-3 significantly at protein level [11]. Previously, PDA-66 was reported to have a depolymerizing effect on tubulin assembly *in vitro* inducing microtubule destabilization in differentiated human neural progenitor cells [12]. However, the effects of PDA-66 and PDA-377 on lymphoma cells have not been characterized before.

Aim of this study was to characterize the influence of PDA-66 and PDA-377 on the two canine B-cell lymphoma cell lines CLBL-1 and CLBL-1M at cellular and molecular level. Due to the similarities in presentation and biologic behavior of lymphomas in dogs and humans, therapeutic protocols of these compounds in dogs could bear high transfer potential to the human disease.

## RESULTS

### PDA-66 and PDA-377 inhibit proliferation and metabolic activity of canine B-cell lymphoma cell lines

PDA-66 demonstrated a strong effect on CLBL-1 and CLBL-1M proliferation. The incubation of CLBL-1 and CLBL-1M with 2.5 μM PDA-66 resulted in a significant decrease in cell count, since cells did not proliferate over the incubation period of 72 h. Cells exposed to 1.0 μM PDA-66 proliferated slower in comparison to the dimethyl sulfoxide (DMSO)-exposed controls. Concentrations below 1.0 μM PDA-66 did not show proliferation-inhibiting effects.

**Figure 1:**
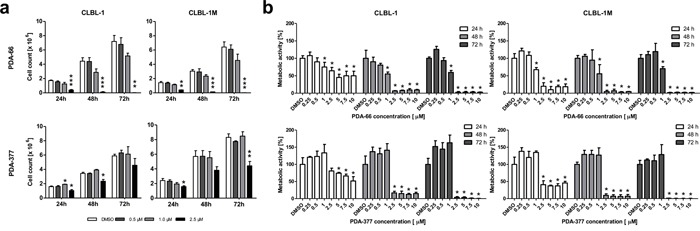
Exposure to PDA-66 and PDA-377 inhibits cell proliferation and metabolic activity in CLBL-1 and CLBL-1M **a.** CLBL-1 and CLBL-1M cells were incubated with different concentrations of PDA-66 and PDA-377 for 24 h, 48 h and 72 h. The proliferation was suppressed significantly at the concentration of 2.5 μM. The diagrams show the mean ± SD of three independent counting experiments. Significance of a treatment effect compared to the DMSO control was determined using student's t-test, *p*<0.05. **b.** CLBL-1 and CLBL-1M cells were incubated with different concentrations of PDA-66 and PDA-377 ranging from 0.25 μM to 10 μM and incubated for 24 h, 48 h and 72 h. Metabolic activity was determined using WST-1 assay. The results are expressed as percentage of the DMSO-treated cells. The diagrams show the mean ± SD of four independent measurements. Significance of a treatment effect compared to the DMSO control was determined using Dunnett's Multiple Comparison Test with a *p* value of < 0.05. *: p<0.05; **: p<0.01; ***: p<0.001.

Application of 2.5 μM PDA-377 led to a significant decrease in proliferation after 24 h and 48 h incubation in CLBL-1, while CLBL-1M showed a significant decrease in proliferation after 24 h and 72 h incubation. The CLBL-1 and CLBL-1M cells treated with 0.5 μM and 1.0 μM PDA-377 proliferated comparable to DMSO-treated control cells (Figure 1a).

A significant dose-dependent effect of PDA-66 and PDA-377 on the metabolic activity could be observed. For both cell lines, PDA-66 showed a significant effect on metabolism, as assessed by the water-soluble tetrazolium (WST-1) assay. At ≥1.0 μM a decrease to ~ 55 − 75 % (depending on time-point) was detected. In contrast, a significant loss was not observed for PDA-377 before increasing the concentration to 2.5 μM. At ≥2.5 μM a loss of metabolic activity was observed after 24 h and was sustained, with almost a complete loss from 48 h onward, in both cell lines with both substances. The detailed concentration/time courses are depicted in Figure 1b. Additional metabolic activity analyses showed that the inhibitory effect of PDA-66 started at 1.5 μM after 48 h of application and at 1.25 μM after 48 h of application (data not shown).

### PDA-66 and PDA-377 induce apoptosis and cell death in canine B-cell lymphoma cell lines

**Figure 2:**
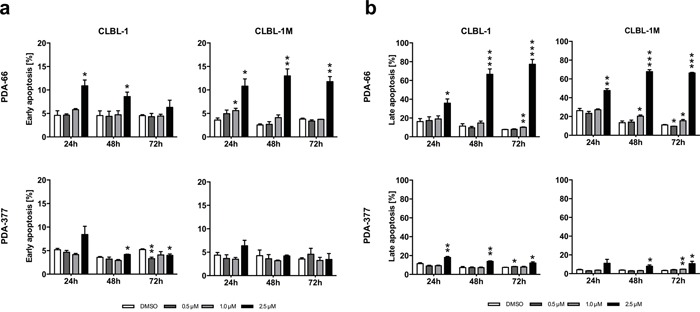
PDA-66 and PDA-377 induce apoptosis CLBL-1 and CLBL-1M cells were exposed to 0.5 μM, 1.0 μM and 2.5 μM PDA-66 and PDA-377 for 24 h, 48 h and 72 h. Analysis of early apoptosis and late apoptosis was performed using flow cytometry after Annexin V FITC and propidium iodide (PI) staining. As a reference DMSO treated cells were analyzed. Rates of early apoptotic (FITC^+^, PI^−^) and late apoptotic/dead (FITC^+^, PI^+^) cells were determined and displayed as the mean ± SD of three independent measurements. **a.** Rate of early apoptotic cells after 24 h, 48 h and 72 h. **b.** Rate of late apoptotic/dead cells after 24 h, 48 h and 72 h. Significance of a treatment effect compared to the DMSO control was determined using student's t-Test, *p*<0.05. *: p<0.05; **: p<0.01; ***: p<0.001.

The effect of PDA-66 and PDA-377 on apoptosis and vitality was analyzed by Annexin V/PI staining 24 h, 48 h and 72 h after PDA application. The distribution of early apoptotic cells (Annexin^+^/PI^−^, Figure 2a) and late apoptotic/dead cells (Annexin^+^/PI^+^, Figure 2b) was determined.

A significant increase of early apoptosis and late apoptotic/dead cells was observed in both cell lines 24 h, 48 h and 72 h after exposure to 2.5 μM PDA-66. After 72 h the application of 2.5 μM PDA-66 late apoptotic/dead cell rates reached 77.3% in CLBL-1 and 66.3 % CLBL-1M. The early apoptotic induction by PDA-377 was less pronounced than by PDA-66. A significant increase was only observed in CLBL-1 cells at 48 h after treatment with 2.5 μM PDA-377. Although the amount of early apoptotic cells in both cell lines increased 24 h after PDA-377 application, the differences to the control cells were not significant. In both cell lines, a significant increase in late apoptotic/dead cells up to 20.0 % was observed 72 h after treatment with 2.5 μM PDA-377 (Supplementary Table 1).

### PDA-66 and PDA-377 cause morphological changes

To verify possible morphological changes caused by PDA-66 and PDA-377, cells were exposed to concentrations ranging from 0.25 μM to 10 μM for 72 h and analyzed by performing life cell imaging. Additionally, cells were treated with 1.5 μM of PDA-66 and 2.5 μM of PDA-377 for 72 h and analyzed by light microscopy after Pappenheim staining.

**Figure 3:**
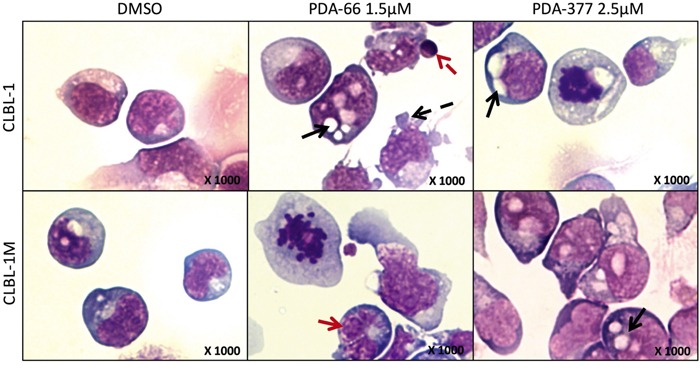
The morphological changes after PDA-66 and PDA-377 treatment CLBL-1 and CLBL-1M cells were incubated with 1.5 μM PDA-66, 2.5 μM PDA-377 and DMSO. Cytospins of treated cells were stained with Pappenheim method after 72 h. Representative pictures are displayed. In DMSO controls, lymphoid round cells show moderately abundant light to dark blue cytoplasm with distinct clear Golgi zones and sometimes distinct vacuolization. The nuclei are single round, indent to cloverleaf-shaped showing coarse chromatin and round eccentric 1-5 nucleoli. In the cells exposed to PDA agents, cells are round to pleomorphic with light to dark blue abundant cytoplasm showing distinct Golgi zones and distinct large vacuolization. Nuclei of round to indent and cloverleaf-shaped (red solid arrow) present with clumped condensed chromatin pattern and rare eccentric round 1-3 nucleoli. Additionally, apoptotic bodies (red dashed arrow), cytoplasmic blebbing (black dashed arrow), increased cytoplasmic and nuclear vacuolization (black solid arrows) and mitotic figures could be observed in analyzed cells.

The two B-cell lymphoma cell lines showed similar morphological changes after PDA application compared to DMSO-treated control cells. Starting at a concentration of 2.5 μM for both PDA derivatives, decreasing cell numbers and an increasing amount of cellular debris and spherically shaped, smaller cells could be observed in contrast to DMSO-exposed cells. With increasing incubation time, the effects on morphology caused by PDA-66 and PDA-377 became more distinct (Supplementary Videos). By Pappenheim staining, the PDA-treated cells exhibited an apoptotic phenomenon. Formation of cytoplasmic blebbing, light to dark blue cytoplasm showing, beside juxtanuclear enlightenment, also distinct large vacuolization, clumped condensed chromatin patterns of round to indented and clover-leaf-shaped nuclei, apoptotic bodies and mitotic figures could be observed in analyzed cells (Figure 3).

### Transcriptomic analyses of PDA-66-treated CLBL-1 and CLBL-1M cells

**Figure 4:**
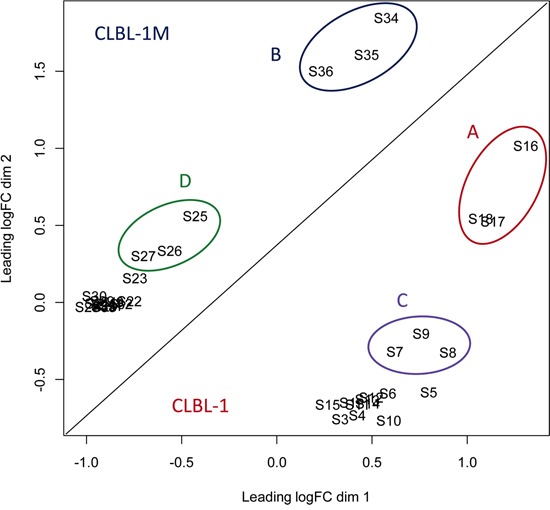
The MDS plot of the RNA-seq data The distances correspond to the differences in the biological coefficient of variation between the samples. The analyzed cells were treated with 1.0 μM and 2.5 μM PDA-66 and DMSO respectively for 12 h and 24 h. Four distinct clusters were observed in MDS plot. The biological replicates form close clusters. **Cluster A**: High-dose (2.5 μM) 24 h treatment CLBL-1; **Cluster B**: High-dose (2.5 μM) 24 h treatment CLBL-1M; **Cluster C**: High-dose (2.5 μM) 12 h treatment CLBL-1; **Cluster D**: High-dose (2.5 μM) 12 h treatment CLBL-1M. **S (1-36)**: Sample names of PDA-66 and DMSO-treated cells.

The multidimensional scaling (MDS) plot of the RNAseq data is given in Figure 4. The high-dose.24 h treatment groups of both cell lines form clearly separated clusters (Cluster A and B), whereas the difference of the high-dose.12 h treatment groups is less pronounced (Cluster C and D). The low-dose treatments did not form clusters separate from the DMSO controls. The lower response to the low-dose treatment is also reflected by the low numbers of differentially expressed genes (FDR < 0.001) (Table 2).

**Table 1:** List of samples used in RNA sequencing

Symbol	Sample name	Symbol	Sample name
**S1**	CLBL-1 DMSO 12h I	**S19**	CLBL-1M DMSO 12h I
**S2**	CLBL-1 DMSO 12h II	**S20**	CLBL-1M DMSO 12h II
**S3**	CLBL-1 DMSO 12h III	**S21**	CLBL-1M DMSO 12h III
**S4**	CLBL-1 PDA-66 1.0 μM 12h I	**S22**	CLBL-1M PDA-66 1.0 μM 12h I
**S5**	CLBL-1 PDA-66 1.0 μM 12h II	**S23**	CLBL-1M PDA-66 1.0 μM 12h II
**S6**	CLBL-1 PDA-66 1.0 μM 12h III	**S24**	CLBL-1M PDA-66 1.0 μM 12h III
**S7**	CLBL-1 PDA-66 2.5 μM 12h I	**S25**	CLBL-1M PDA-66 2.5 μM 12h I
**S8**	CLBL-1 PDA-66 2.5 μM 12h II	**S26**	CLBL-1M PDA-66 2.5 μM 12h II
**S9**	CLBL-1 PDA-66 2.5 μM 12h III	**S27**	CLBL-1M PDA-66 2.5 μM 12h III
**S10**	CLBL-1 DMSO 24h I	**S28**	CLBL-1M DMSO 24h I
**S11**	CLBL-1 DMSO 24h II	**S29**	CLBL-1M DMSO 24h II
**S12**	CLBL-1 DMSO 24h III	**S30**	CLBL-1M DMSO 24h III
**S13**	CLBL-1 PDA-66 1.0 μM 24h I	**S31**	CLBL-1M PDA-66 1.0 μM 24h I
**S14**	CLBL-1 PDA-66 1.0 μM 24h II	**S32**	CLBL-1M PDA-66 1.0 μM 24h II
**S15**	CLBL-1 PDA-66 1.0 μM 24h III	**S33**	CLBL-1M PDA-66 1.0 μM 24h III
**S16**	CLBL-1 PDA-66 2.5 μM 24h I	**S34**	CLBL-1M PDA-66 2.5 μM 24h I
**S17**	CLBL-1 PDA-66 2.5 μM 24h II	**S35**	CLBL-1M PDA-66 2.5 μM 24h II
**S18**	CLBL-1 PDA-66 2.5 μM 24h III	**S36**	CLBL-1M PDA-66 2.5 μM 24h III

**Table 2:** Significant differentially expressed genes in PDA-66-treated lymphoma cells (versus DMSO-control)

Cell line	Treatment	Count of differentially expressed genes (FDR<0.001)^a)^
CLBL-1	1.0 μM 12h	3
CLBL-1M	1.0 μM 12h	1
CLBL-1	1.0 μM 24h	33
CLBL-1M	1.0 μM 24h	1
CLBL-1	2.5 μM 12h	170
CLBL-1M	2.5 μM 12h	235
CLBL-1	2.5 μM 24h	2191
CLBL-1M	2.5 μM 24h	4780

a) FDR: false discovery rate.

The high-dose treatment resulted in higher numbers of differentially expressed genes, which was more pronounced in the CLBL-1M cells at both time points (Table 2). The response after 12 h did not reach statistical significance for any transcript, when CLBL-1 and CLBL-1M were compared, suggesting that the early effects of PDA-66 are similar in both cell lines.

Nevertheless, the comparison of the high-dose.24 h groups yielded 1703 genes that react differently between the two cell lines. Most significantly overrepresented genes in this set are ribosomal proteins, which are transcribed at higher levels in CLBL-1M compared to CLBL-1 cells (corrected for control levels). Significantly deregulated pathways in the high-dose.24 h treatment groups compared to the DMSO exposed controls identified by KEGG analyses are given in Table 3.

**Table 3:** Significantly deregulated KEGG pathways in the high-dose 24 h treatment groups

KEGG Pathway	CLBL-1 2.5 μM 24 h		CLBL-1M 2.5 μM 24 h	
	Count (gene)	FDR^a)^	Count (gene)	FDR
Ribosome	49	2.42E-18	65	3.02E-15
Cell cycle	39	5.03E-08	54	3.49E-05
Oxidative phosphorylation	35	6.66E-05	57	1.47E-05
DNA replication	15	0.00222	20	0.0163
Oocyte meiosis	26	0.0455		
RNA degradation			32	1.20E-04

a) FDR: false discovery rate.

### Early deregulated genes and associated pathways

**Figure 5:**
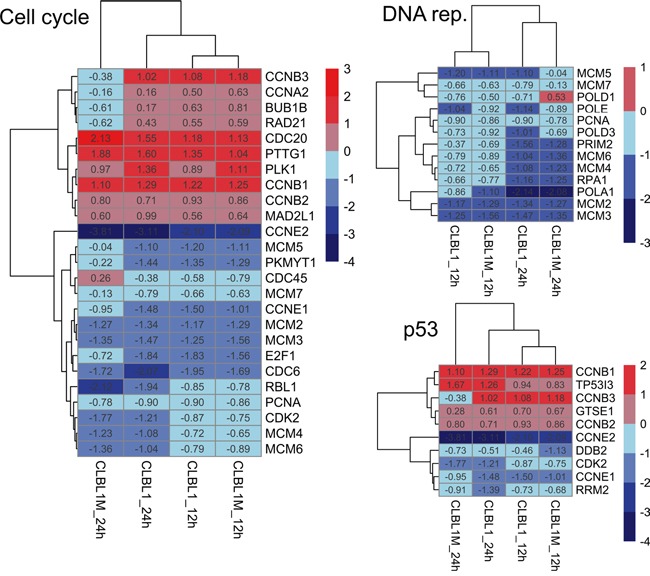
Heatmap of early (12 h) reacting genes in all high-dose treatment groups Genes with significantly different expression (FDR<0.001) in any of the high-dose treatments compared to controls were selected and displayed as a heatmap with euclidean distance clustering. Numbers given for each gene are the fold changes expressed as logarithmized ratios (base 2).

**Figure 6:**
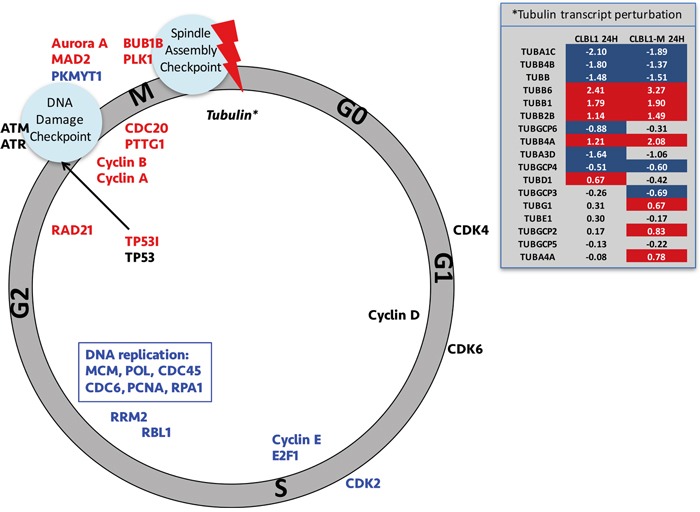
Schematic overview of the PDA-66 effect on cell cycle Schematic overview of the PDA-66 effect on cell cycle-related gene expression. Genes printed in blue are downregulated and genes printed in red are upregulated. Cell cycle-related kinases are printed on the outside of the circle. Genes that are expressed during the G2 phase of the cell cycle are upregulated in the PDA-66 cell cultures as compared to controls, while G1 and S-phase associated genes are significantly downregulated. The expression pattern together with the perturbation in tubulin isoform transcript abundance (insert) suggests activation of the spindle assembly checkpoint (red bolt). Insert: Tubulin isoforms and associated complexes are listed and log-fold changes are given for the two cell lines after 24 h treatment with 2.5 μM PDA-66. Values highlighted in red or blue indicate significant higher or lower abundances, respectively. Greyed values are non-significant changes (FDR>0.05).

The early effects (12 h) of PDA-66 involved deregulation of 170 genes in CLBL-1 cells and 235 genes in CLBL-1M cells. Both 12h.high-dose treatment groups showed highly significant enrichment of genes mapping to the KEGG pathways: cell cycle, DNA replication and p53 signaling (Table 4). The fold-changes of the early affected genes in these pathways are displayed as heatmaps for all four high-dose treatment groups in Figure 5 and show consistent directions of changes in the 12 h treatment groups. An overview of this drug-induced gene regulation in the context of the mammalian cell cycle is displayed in Figure 6. The results of the transcriptome analysis suggest a cell cycle arrest putatively in late M-phase, likely involving the spindle assembly checkpoint.

**Table 4:** Significantly deregulated KEGG pathways in the high-dose 12 h treatment groups

KEGG Pathway	CLBL-1 2.5 μM 12 h		CLBL-1M 2.5 μM 12 h	
	Count (gene)	FDR^a)^	Count (gene)	FDR
Cell cycle	22	6.06E-20	25	8.78E-22
DNA replication	11	1.51E-10	12	4.23E-11
p53 signaling pathway	9	6.48E-05	10	2.81E-05

a) FDR: false discovery rate.

## DISCUSSION

Herein, the effects of PDA-66 and PDA-377 on canine lymphoma cell lines were investigated for the first time. Different dosages and incubation times were compared to DMSO-treated control cultures. The DMSO concentration (≤ 0.1 %) in the control groups was chosen to be equivalent to the highest DMSO concentration of the PDA group in order to compensate for putative solvent effects. Nevertheless, such low concentrations of DMSO are described to cause no cell toxicity [13, 14].

Consistent with previous findings, PDA-66 and PDA-377 led to a significant decrease in cell count, metabolic activity, and induced anti-proliferative effects in canine B-cell lymphoma cells. Furthermore, an induction of apoptosis and late apoptotic/dead cells was detected in the exposed lymphoma cells. Compared with PDA-377, PDA-66 showed a stronger cytotoxic effect inducing death of all lymphoma cells after 48 h at a concentration of 2.5 μM. In fact, inhibition to 70 % proliferation and apoptosis of 10 % was observed at 24 h post 2.5 μM PDA-66 application. Although inhibitory effects of 1.0 μM PDA-66 on cell proliferation and metabolic activity were also observed, the cells maintained a comparable growth rate to DMSO-treated control. Previous studies on human ALL cell lines have shown a significant inhibition of proliferation starting at a concentration of 0.5 μM PDA-66 [11]. In the ReNcell VM human neural progenitor cell line (hNPC), proliferation can be stopped by application of 3 μM PDA-66 while human neuroblastoma cells (SH-SY5Y) with an IC_50_ of 8.48 μM and lung cancer cells (A549) with an IC_50_ of 4.97 μM were less sensitive to PDA-66 [12].

Comparative transcriptome analysis was performed for the more potent compound (PDA-66) at 12 h and 24 h post application of dosages of 1.0 μM and 2.5 μM. Low-dose-treated groups did not show major gene expression differences compared to controls while high-dose application induced distinct effects. Early modulated genes and pathways (12 h PDA-66 high-dose) mapped to three main pathways: cell cycle, DNA replication and p53 signaling pathway. However, the gene sets assigned to the p53 and cell cycle pathway largely overlap. The effect of PDA-66 on cell cycle and DNA replication was more pronounced at 24 h, indicating a time-dependent effect in both cell lines. Minor differences in transcript abundance of ribosomal proteins were detected between the CLBL-1 and CLBL-1M 24 h high-dose groups. The deregulated pathways (Table 3) showed slightly different levels of significance. These observed subtle differences only seen in the 24 h high dose reaction of the two cell lines could be caused by clonal selection as the cell line CLBL-1M was generated by *in vivo* inoculation of CLBL-1. However, the general response of both cell lines and the major addressed pathways are comparable indicating a common acting mechanism of the compounds.

Expression analyses showed that especially genes, for which peak transcription is described in late G2/M phase are significantly elevated in the treated cells, while G1 and S-phase-associated genes were significantly depleted (Figure 6) [15]. This observation strongly suggests that PDA-66 induces a cell cycle block, which arrests the cells and ultimately leads to apoptosis. Three cell cycle checkpoints are known in mammalian cells: the G1 checkpoint, the G2-M DNA damage response checkpoint (DDR) and the spindle assembly checkpoint (SAC). Though our RNAseq data clearly excluded an arrest at G1 phase, we detected the transcriptional enrichment of genes related to both DDR and SAC. While the enrichment of TP53I3 (tumor protein p53 inducible protein) transcripts points to an activation of TP53 mediated DDR, the enriched MAD2, CDC20 and BUB1B transcripts represent key components of the SAC [16, 17].

Interestingly, significant differential transcript abundance for tubulin isoforms was solely detected in the 24 h treatment groups, and different isoforms showed different directions of change (Figure 6 insert). It has been described previously that PDA-66 has a depolymerizing effect on tubulin assembly *in vitro* and acts as microtubule-destabilizing agent by its indole core [12]. Furthermore, an autoregulatory mechanism of tubulin transcription has been proposed whereby tubulin mRNA expression/stability is negatively regulated by soluble unpolymerized tubulin dimers accumulating within the cell [18]. Taken together the enrichment of G2/M phase-specific transcripts and the later perturbation of tubulin isoform expression suggest a cell cycle arrest at the SAC rather than DDR. Such a SAC arrest was recently also reported on a different indole-containing anti-cancer agent [19].

In conclusion, we have shown that PDA-66 as well as PDA-377 significantly inhibit the proliferation and metabolic activity of two canine lymphoma cell lines and induce apoptosis as previously demonstrated for human neuroblastoma, lung carcinoma and ALL cells, also with dose-dependent G2 arrest [11, 12]. Our results show that PDA-66 possesses potential anti-cancer activity through SAC activation.

The characterization of PDA-induced effects on canine lymphoma cells provides a basis for further *in vivo* studies. Early pharmacokinetic studies in mice revealed that i.p. application of 100 mg/kg PDA-66 once daily was well tolerated. One hour after injection plasma concentrations between 4 and 7 μmol/L were achieved rapidly decreasing exponentially to nearly zero after five hours (data not shown).

However, an application in dogs with spontaneously occurring lymphoma could allow evaluating the compound in presence of an immune system. Consequently, single or combination therapy with PDA-66 in dogs can provide valuable information before compound testing in humans.

## MATERIALS AND METHODS

### Arylindolylmaleimides PDA-66 and PDA-377

PDA-66 and PDA-377 were synthesized and kindly provided by the Leibniz Institute for Catalysis (Rostock, Germany). Chemical structures of both substances and the analogue SB-216763 were described previously [11, 12].

The substances were dissolved in DMSO (AppliChem, Darmstadt, Germany). The stock solutions (10 mM) were stored at −20°C. For experimental use the drugs were freshly prepared from stock solution.

### Cell lines and culture condition

Two canine B-cell lymphoma cell lines named CLBL-1 and CLBL-1M established by the authors were used. CLBL-1 was derived from a fine needle aspirate of an 8 year-old Bernese Mountain Dog with stage IV diffuse large cell lymphoma [5]. The daughter cell line CLBL-1M was generated by injecting CLBL-1 cells into Rag2^−/−^γc^−/−^mice. The emerging tumorous material was isolated, cultivated and the lymphoma cell line was established. CLBL-1M allowed characterizing the influence of *in vivo* inoculation of CLBL-1 cells evaluating the stability and comparability of CLBL-1-generated *in vitro* and *in vivo* models. Comparison of flow cytometric, cellular, molecular and chromosomal markers revealed strong conservative character of both cell lines [20]. Additional, comparative genomic sequencing revealed high genomic stability with one exception, a focal deletion at CFA32: 5Mbp-7.5Mbp which is exclusively present in CLBL-1 (unpublished data).

CLBL-1 and CLBL-1M cells were cultivated in RPMI 1640 medium (Biochrom, Berlin, Germany) supplemented with 20 % heat-inactivated fetal bovine serum (FBS) (Biochrom) and 2 % penicillin/streptomycin (Biochrom) at 37°C in a humidified atmosphere of 5 % CO_2_ in T75-tissue culture flasks placed in upright position (TPP, Trasadingen, Switzerland).

### WST-1 proliferation assay

CLBL-1 and CLBL-1M cells were seeded at a density of 5 × 10^4^ cells/well in 96-well plates with 150 μl cell culture medium containing different concentrations of PDA-66 and PDA-377 (0.25 − 10 μM). Control cells were cultured in medium containing 0.1 % (v/v) of DMSO. Cells treated with each PDA concentration were plated in four replicates. All cells were incubated for 24 h, 48 h and 72 h. The WST-1 (Roche, Mannheim, Germany) analysis was performed as described before [11]. The absorbance at 450 nm and a reference wavelength at 620 nm were determined using the Multi-Mode Reader Synergy 2 (BioTek Instruments, VT, USA).

### Cell count analysis

CLBL-1 and CLBL-1M cells were seeded at a density of 1×10^6^ cells in 3 ml of cell culture medium in 12-well plates. Cells were treated with 0.025 % DMSO, 0.5 μM, 1.0 μM and 2.5 μM PDA-66 and PDA-377, respectively. The number of viable cells was determined 24 h, 48 h and 72 h after DMSO, PDA-66 and PDA-377 treatment by trypan blue staining using a hemocytometer. Experiments were performed in biological triplicates.

### Analysis of apoptosis and late apoptotic/dead cells

Apoptosis and late apoptotic/dead cell were analyzed by staining with Annexin V FITC (BD Biosciences, Heidelberg, Germany) and Propidium iodide (PI) (Sigma Aldrich, St. Louis, USA) according to the manufacturer's protocol. The apoptosis and late apoptotic/dead cells rates were determined by flow cytometry using a FACSCalibur^TM^ (BD Biosciences, Heidelberg, Germany).

After cell count analysis, 1 × 10^6^ CLBL-1 and CLBL-1M cells were harvested and washed twice (185 xg, 10 min, 4°C) with PBS, then resuspended in 100 μl of Annexin binding buffer (BD Biosciences, Heidelberg, Germany). Five μl of Annexin V FITC were added and incubated for 15 min at room temperature. Cell suspensions were adjusted to a final volume of 500 μl with binding buffer and stained with PI (0.6 μg/ml) immediately before measurement. Unstained and single stained cells (0.025 % DMSO-treated) were included in each experiment as controls. The gained data were analyzed using BD CellQuest software (BD Biosciences, Heidelberg, Germany).

### Live cell imaging

CLBL-1 and CLBL-1M cells were seeded at a density of 5 × 10^4^ cells/well in a 96-well plate with 150 μl cell culture medium and treated with 0.1 % DMSO and different concentrations of PDA-66 and PDA-377 (0.25 − 10 μM). Cells treated with each PDA concentration were plated with three biological replicates. The imaging was performed for 96 h with PDA treatment at 37°C in a humidified atmosphere of 5 % CO_2_ using the Leica DMI6000 B Inverted Microscope (Leica Microsystems, Wetzlar, Germany). Simultaneously, a picture of each attempt was captured every 15 min during the whole incubation time and single pictures were combined to a Live cell imaging video.

### Pappenheim staining

Cells were treated with DMSO, 1.5 μM PDA-66 and 2.5 μM PDA-377 for 72 h. Glass slides were prepared with 5 × 10^4^ cells with Cytospin 3 centrifuge (Shandon, Frankfurt/Main, Germany). Briefly, slides were stained in May-Grünwald working solution (Merck, Darmstadt, Germany) for 6 min, rinsed with tap water, then stained 20 min in Giemsa working solution (Merck, Darmstadt, Germany) and rinsed thoroughly with tap water. The slides were air dried at room temperature before analysis. To evaluate morphological changes of the cells slides were analyzed by EVOS® XL Core Imaging System (AMG, Washington, USA).

### Transcriptomic analyses

Due to the more pronounced effect of PDA-66 at cellular level, the transcriptome characterization was only performed for PDA-66.

CLBL-1 and CLBL-1M cells were treated with 0.025 % DMSO, 1.0 μM (low-dose) and 2.5 μM (high-dose) PDA-66. At 12 h and 24 h, cells were harvested and total RNA was extracted using the miRNeasy Mini Kit (Qiagen, Hilden, Germany) according to the manufacturer's protocol. Sample names are shown in Table 1. Each treatment condition was prepared and sequenced in triplicates. For the preparation of sequencing libraries, 2 μg total RNA with RNA integrity numbers > 8 were used. Poly-A RNA was enriched and ligated to sequencing adapter using the NEBNext Ultra RNA preparation Kit (New England Biolabs, Ipswich, USA) according to the manufacturer's protocols. Sequencing was conducted on an Illumina NextSeq500 (Illumina, San Diego, USA) as single reads with 75 bp length. A total of 480 million reads were generated, which were mapped to the canine genome (Broad CanFam3.1/canFam3, Sep. 2011) using Burrows-Wheeler Aligner (BWA) [21]. Read counts were generated for annotated genes (EMBL gene ID nomenclature) using the R package GAGE [22]. Annotations referring to non-protein coding RNAs were censored from the final read count list.

### Bioinformatic analyses and statistics

Significant differences of metabolic activity between treatment and the DMSO control were calculated using Dunnett's multiple comparison test. The statistical analyses of cell count and apoptosis/necrosis were done using student's *t-test* (GraphPad Software, La Jolla, USA). Results are shown as mean ± standard deviation (SD). Differences were considered statistically significant if * *p*< 0.05.

Differential gene expression analysis from RNAseq data was conducted using the BioconductorR package edgeR [23]. Genes with less than 1 read count per million in more than 18 samples were censored. On average 4.5 million (STDEV: 745,000) mapped reads per sample were used for differential gene expression analyses. Each cell line/treatment group (n=3) was compared to the respective mock-treated (DMSO) control cells (n=3). Treatment groups were high-dose.12 h, high-dose.24 h, low-dose.12 h, low-dose.24 h. After multidimensional scaling and plotting of the data one CLBL-1 DMSO-treated control sample was identified as outlier and was censored, so that the CLBL-1-12h-DMSO group consisted of only two independent samples. In addition to the comparison of each treatment group within the two cell lines, genes that responded differently to 12 h and 24 h high-dose treatment in CLBL1 versus CLBL-1M were identified using the generalized linear model functionality of edgeR [23]. Genes with a false discovery rate-corrected *p*-value (FDR) of less than 0.001 were considered as significantly deregulated and were mapped to the Kyoto Encyclopedia of Genes and Genomes (KEGG) pathways for functional enrichment analyses using the Database for Annotation, Visualization and Integrated Discovery (DAVID) Functional Annotation Tool (http://david.abcc.ncifcrf.gov).

## SUPPLEMENTARY TABLE AND VIDEOS






