# Supplementary material for: Characterization of the novel indolylmaleimides' PDA-66 and PDA-377 effect on canine lymphoma cells
Source: Oncotarget. 2016 May 12;7(23):35379–89. doi: 10.18632/oncotarget.9297 (PMC5085236; doi:10.18632/oncotarget.9297)
Supplement: Supplementary file 1 [file oncotarget-07-35379-s001.pdf]

Characterization of the novel indolylmaleimides' PDA-66 and PDA-377 effect on canine lymphoma cells

SUPPLEMENTARY TABLE AND VIDEOS

Supplementary Table 1: PDA-66 and PDA-377 induce apoptosis and cell death in canine B-cell lymphoma cell lines

		Early apoptosis (%)				Late apoptosis (%)			
		**DMSO**	**PDA-66**			**DMSO**	**PDA-66**		
		**0.0025%**	**0.5 µM**	**1.0 µM**	**2.5 µM**	**0.0025%**	**0.5 µM**	**1.0 µM**	**2.5 µM**
CLBL-1	24h	4.58 ± 1.69	4.66 ± 0.46	5.81 ± 0.41	10.88 ± 1.76	16.17 ± 5.54	17.33 ± 6.89	19.01 ± 5.77	35.82 ± 7.72
	48h	4.55 ± 1.72	4.38 ± 1.90	4.73 ± 1.44	8.58 ± 1.66	11.37 ± 4.43	9.59 ± 2.46	14.66 ± 3.66	66.49 ± 10.06
	72h	4.53 ± 0.37	4.30 ± 1.23	4.44 ± 0.69	6.29 ± 2.63	7.70 ± 0.37	7.90 ± 1.00	10.14 ± 0.65	77.28 ± 8.89
CLBL-1M	24h	3.60 ± 0.75	4.94 ± 1.35	5.60 ± 0.86	10.80 ± 2.70	26.27 ± 3.99	23.32 ± 3.54	27.16 ± 1.74	47.62 ± 3.41
	48h	2.55 ± 0.37	2.68 ± 0.96	4.10 ± 1.02	12.98 ± 2.60	13.42 ± 3.16	14.05 ± 3.71	20.30 ± 2.35	67.72 ± 3.68
	72h	3.81 ± 0.31	3.38 ± 0.44	3.75 ± 0.04	11.77 ± 1.86	11.07 ± 0.80	9.63 ± 0.09	15.41 ± 2.48	66.33 ± 1.20
		**DMSO**	**PDA-377**			**DMSO**	**PDA-377**		
		**0.0025%**	**0.5 µM**	**1.0 µM**	**2.5 µM**	**0.0025%**	**0.5 µM**	**1.0 µM**	**2.5 µM**
CLBL-1	24h	5.18 ± 0.49	4.65 ± 0.69	4.16 ± 0.43	6.71 ± 0.69	11.37 ± 1.90	8.99 ± 1.30	9.22 ± 1.02	18.12 ± 1.65
	48h	3.57 ± 0.30	3.20 ± 0.74	2.91 ± 0.34	4.18 ± 0.12	7.27 ± 1.92	7.20 ± 1.25	7.20 ± 1.14	13.85 ± 0.96
	72h	5.24 ± 0.25	3.30 ± 0.55	4.09 ± 1.25	4.00 ± 0.49	7.42 ± 0.43	8.27 ± 0.31	7.83 ± 1.38	12.07 ± 2.22
CLBL-1M	24h	4.36 ± 0.99	3.65 ± 1.37	3.51 ± 0.64	6.37 ± 2.02	4.22 ± 0.81	3.01 ± 0.44	3.60 ± 0.37	11.00 ± 7.40
	48h	4.26 ± 2.11	3.60 ± 1.53	3.19 ± 0.22	4.20 ± 0.33	3.70 ± 0.57	2.92 ± 0.53	3.12 ± 0.36	7.97 ± 2.32
	72h	3.55 ± 0.45	4.57 ± 2.18	3.24 ± 1.15	3.45 ± 1.79	3.33 ± 0.42	4.04 ± 0.71	4.65 ± 0.21	10.63 ± 4.47

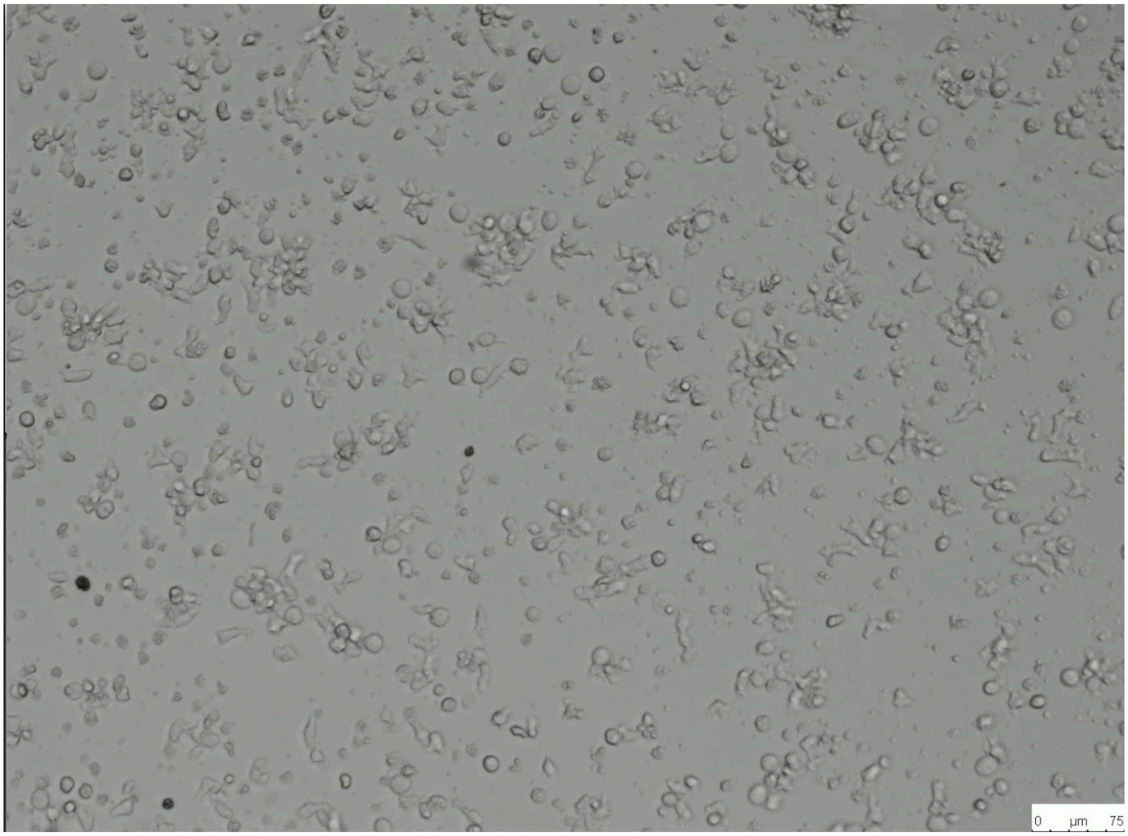

Supplementary Video 1: Live cell imaging of CLBL-1M cells after PDA-66 application. CLBL-1M cells were cultured with 2.5 µM PDA-66. The cellular reaction was monitored for 36h. Scale bar, 75 µm.

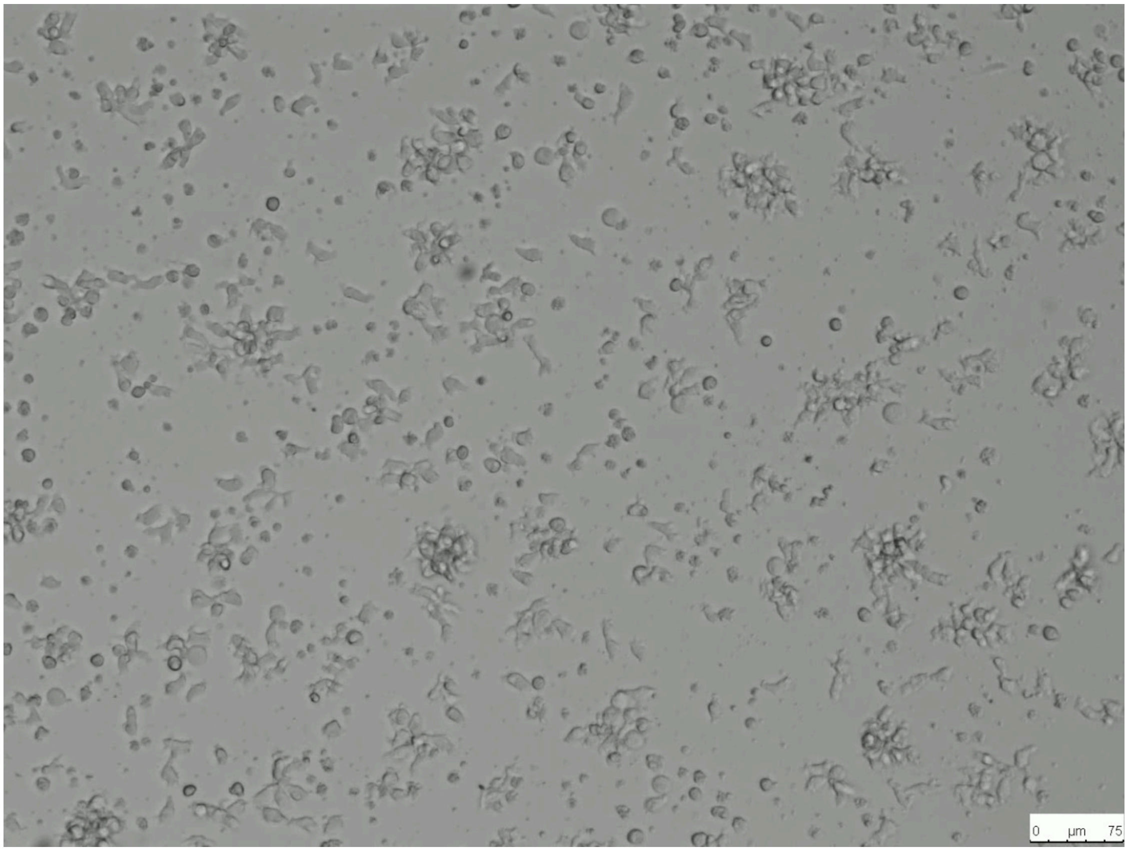

Supplementary Video 2: Live cell imaging of CLBL-1M cells after DMSO application. CLBL-1M cells were cultured with 0.1 % DMSO. The cellular reaction was monitored for 36h. Scale bar, 75 µm.
